# Supplementary material for: Biomonitoring in the Anthropocene: Urban estuary environmental DNA tracks marine fish, terrestrial wildlife, and human diet
Source: PLoS One. 2026 Apr 29;21(4):e0332676. doi: 10.1371/journal.pone.0332676 (PMC13127899; doi:10.1371/journal.pone.0332676)
Supplement: S1 Fig — (PDF) [file pone.0332676.s011.pdf]

5′- TGTAAAACGACGGCCAGTATATACATGCAAGTATCCGCACCCCAGTGTA

AATGCCCTTAATGTCTCCCCATTAGACAAAAGGAGCAGGTATCAGGCACACTATCTATGTAGCCCAAAAC

GCCTTGCTTAGCCACACCCCCACGGGTACTCAGCAGTAATTAACATTAAGCAATAAGTGTAAACTTGACT

TAGCCATGGCGACCCCCAAGGGT**C**GGTAAA**A**CT**C**GTGCCAGCCACCGCGGTCACACAAGAGACCCAAACT

AACAGTAGCCGGCGTAAAGAGTGGTAATATGTTATCCAACCAAATTAAGACCCAAACACAGCTAAGCTGT

CACAAGCCCAAGATGTTCCTAAAATCACCATAAAAATGGTCTTAACACCCACGATCAATTTCACCCCACG

AAAGCTAAGGCACAAACTGGGATTAGATACCCCACTATGCTTAGCCTTAAATCATGATACTTACCCCACC

TAAGTATCCGCCCGAGAACTACGAGCACAAACGCTTAAAACTCTAAGGACTTGGCGGTGCCCTAAACCCA

CCTAGAGGAGCCTGTTCTATAATCGATAATCCACGATTCACCCAACCACCCCTTGCCATGCAGCCTACAT

ACCGCCGTCGCCAGCCCGCCTCATGAGAGAACAATAGCGAGCACAATAGCCCACCCGCTAACAAGACAGG

TCAAGGTATAGCATATGGAGTGGAAGAAATGGGCTACATTTTCTAACATAGAATACACACGAAAGAGGAT

AT**GTCATAGCTGTTTCCTG -3′**

S1 Fig. Gene block spike-in standard. Based on *Struthio camelus* mitochondrion, GenBank MN356148. As compared to reference sequence, includes M13 tails, modified bases at MiFish-U-F site (changes vs reference sequence in bold). Green = MiFish-U-F/R2 primer binding sites (blue, extra base added to MiFish-U R primer to reduce bacterial amplification). Yellow = Riaz primer binding sites. The Riaz forward primer site overlaps MiFish-U-F/R2 reverse primer site.
