# Supplementary material for: Biomonitoring in the Anthropocene: Urban estuary environmental DNA tracks marine fish, terrestrial wildlife, and human diet
Source: PLoS One. 2026 Apr 29;21(4):e0332676. doi: 10.1371/journal.pone.0332676 (PMC13127899; doi:10.1371/journal.pone.0332676)
Supplement: S2 Fig — (PDF) [file pone.0332676.s012.pdf]

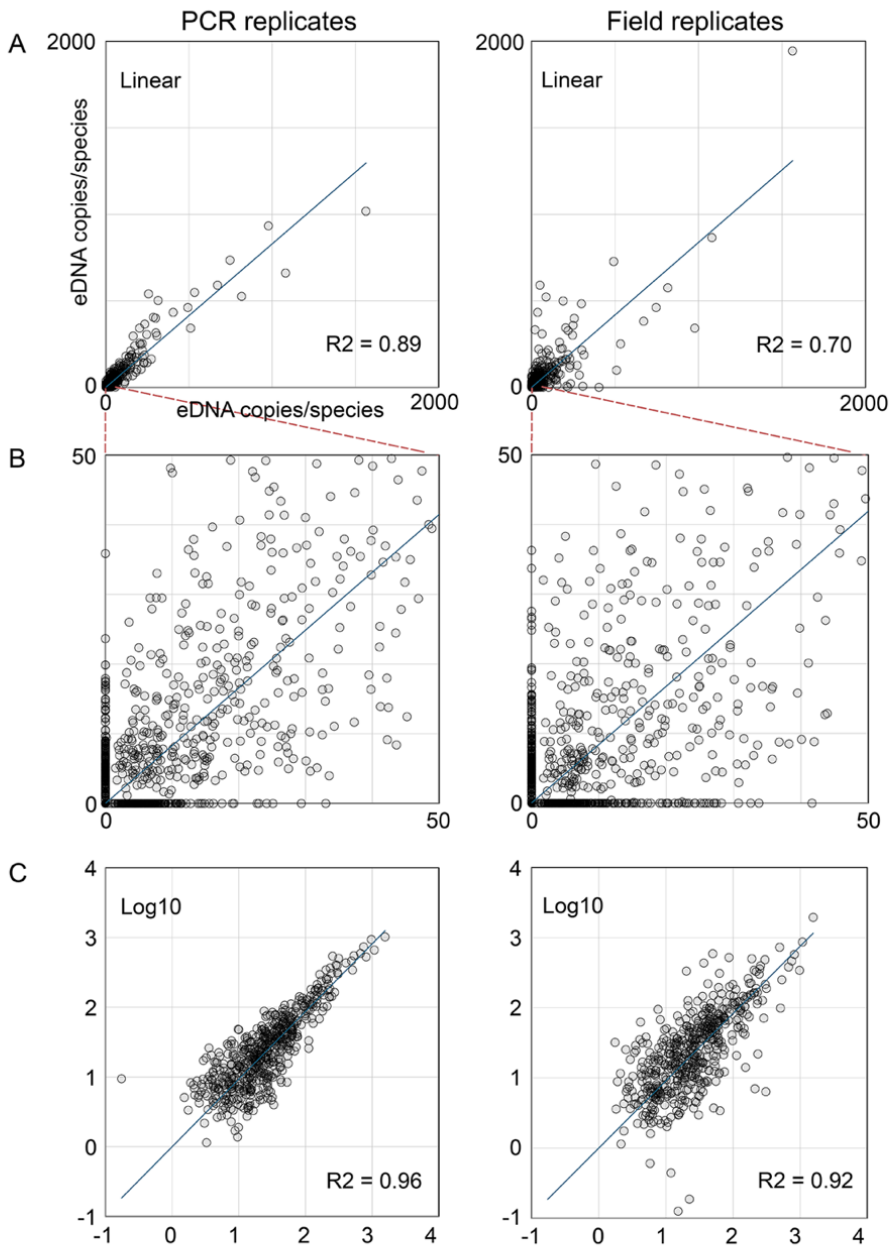

S2 Fig. Reproducibility of individual PCR and field replicates. Each point represents one local marine fish species in one pair of replicate PCRs. Values expressed as eDNA copies per PCR (not per liter). A) Linear scale complete dataset. B) Linear scale subset 0-50 copies/species. Note detections below about 10 copies/PCR frequently not detected in replicate. C) Log scale replicated detections.
