# Supplementary material for: Biomonitoring in the Anthropocene: Urban estuary environmental DNA tracks marine fish, terrestrial wildlife, and human diet
Source: PLoS One. 2026 Apr 29;21(4):e0332676. doi: 10.1371/journal.pone.0332676 (PMC13127899; doi:10.1371/journal.pone.0332676)
Supplement: S3 Fig — (PDF) [file pone.0332676.s013.pdf]

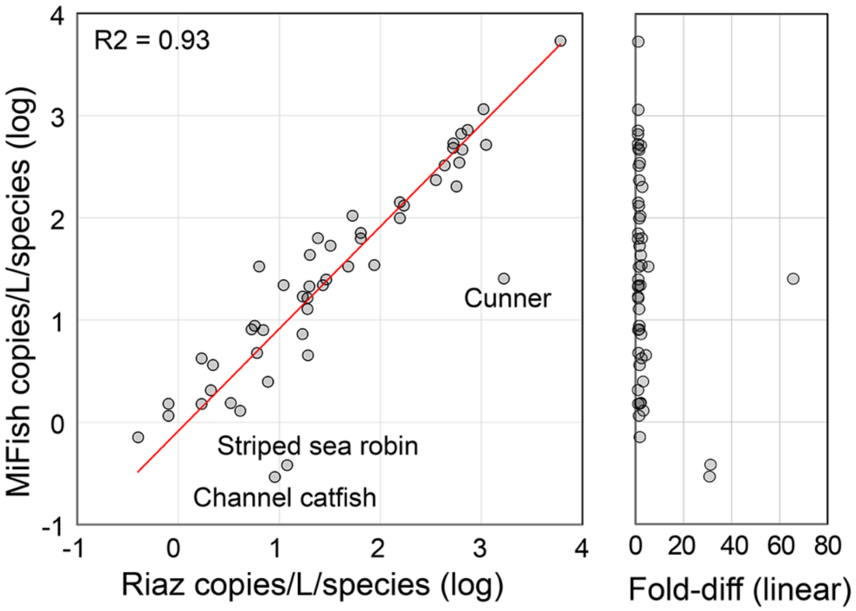

S3 Fig. MiFish-U-F/R2 vs Riaz pooled copies/L. Each point represents one species in a set of pooled PCR replicates (64 PCRs/set).
