# Supplementary material for: Biomonitoring in the Anthropocene: Urban estuary environmental DNA tracks marine fish, terrestrial wildlife, and human diet
Source: PLoS One. 2026 Apr 29;21(4):e0332676. doi: 10.1371/journal.pone.0332676 (PMC13127899; doi:10.1371/journal.pone.0332676)
Supplement: S4 Fig — (PDF) [file pone.0332676.s014.pdf]

ACCESSION	SPECIES	GCCGGT-AAAACTCGTGCCAG	ACAAACTGGGATTAGATACCCCACTATG	CTAGAGGAGCCTG-TTCTA
NC32715	Acipenser brevirostrum	....	....	....
NC28290	Acipenser oxyrinchus	....	....	....
NC37017	Alosa aestivalis	....	....	....
NC37016	Alosa mediocris	....	....	....
PV624612	Alosa pseudoharengus	....	....	....
NC14690	Alosa sapidissima	....	....	....
PV662064	Aluterus schoepfii	....	....	....
NC28530	Ammodytes americanus	....	....	....
PV642271	Anchoa hepsetus	.T...................	....	....
NC82550	Anchoa mitchilli	....	....	....
NC6547	Anguilla rostrata	....	....	....
NC83080	Bothus robinsi	.......G.............	....	....
NC14266	Brevoortia tyrannus	....	....	....
OR482497	Caranx hippos	....	....	....
NC82556	Caranx latus	....	....	....
PV710296	Centropristis striata	....	....	................G.G
PV742855	Chaetodon ocellatus	.T...................	....	....
NC83038	Citharichthys arctifrons	....	....	....
NC9577	Clupea harengus	....	....	....
NC83079	Conger oceanicus	...........T.........	.............T..............	....
OP056988	Cynoscion regalis	.......T.............	....	....
NC8107	Dorosoma cepedianum	....	....	....
OP056804	Etropus microstomus	....	....	..................G
NC12312	Fundulus heteroclitus	......C..............	....	....
NC2081	Gadus morhua	....	....	....
OP035221	Gobiesox strumosus	.T...................	....	....
OP057012	Gobiosoma bosc	....	....	....
OP056822	Gobiosoma ginsburgi	....	....	....
NC22722	Hippocampus erectus	....	....	....
OP035308	Hypleurochilus geminatus	....	....	....
OP056806	Hypsoblennius hentz	....	....	....
NC3489	Ictalurus punctatus	...........T.........	....	....
PV276250	Leiostomus xanthurus	....	....	....
NC15244	Mallotus villosus	.......T..T..........	....	.................-.
OP056990	Membras martinica	....	....	...............C...
MH538898	Menidia menidia	....	....	....
NC88009	Menticirrhus americanus	A....................	....	....
OP056993	Menticirrhus saxatilis	....	....	....
OR499725	Merluccius bilinearis	......A..TG..........	....	....
PV624571	Microgadus tomcod	....	....	....
PV276251	Micropogonias undulatus	.......T.............	....	....
KU641485	Morone americana	..........T..........	....	...............C...
NC14353	Morone saxatilis	..........T..........	....	...............C...
NC3182	Mugil cephalus	..........T..........	....	....
PV339948	Myoxocephalus aenaeus	....	....	....
OP035321	Myrophis punctatus	..........C..........	....	....
MT103916	Myzopsetta ferruginea	....	....	....
PP032991	Ocyurus chrysurus	.T...................	....	....
NC83093	Ophidion marginatum	...........T.........	....	...............C...
PV642264	Opisthonema oglinum	....	....	....
OP057095	Opsanus tau	.T.....C...T.........	................C...........	................TCT
OP056947	Orthopristis chrysoptera	....	................G...........	....
NC29476	Paralichthys dentatus	....	....	....
PV710544	Peprilus triacanthus	....	....	....
NC1626	Petromyzon marinus	..T.......C..........	......C...............G.....	...............C...
NC52755	Pholis gunnellus	....	....	....
OP057004	Pogonias cromis	.......T.............	....	....
OR911369	Pomatomus saltator	....	....	..................G
PV670486	Prionotus carolinus	....	....	....
NC83024	Prionotus evolans	.......T..TT.........	............C...............	....
PV691859	Pseudopleuronectes americanus	....	....	....
NC88021	Pseudupeneus maculatus	....	................G...........	.............T.....
NC13724	Scomber colias	....	....	..............C....
NC6398	Scomber scombrus	....	....	..............C....
OP056995	Scomberomorus maculatus	....	....	....
OP056991	Scophthalmus aquosus	....	....	....
PV720037	Selar crumenophthalmus	....	....	....
NC16870	Seriola dumerili	....	....	....
OP056994	Sphoeroides maculatus	A....................	....	....
OR582685	Stenotomus chrysops	....	....	....
OP057094	Strongylura marina	...........A.........	....	...........C...C...
NC83036	Symphurus plagiusa	.TT..................	....	...............C..G
OK172580	Syngnathus fuscus	....	....	....
NC83026	Synodus foetens	.....C.C.............	....	....
PV329700	Tautoga onitis	..........T..........	....	....
PV276249	Tautogolabrus adspersus	..........T..........	C...........................	....
OP056886	Trachinotus falcatus	....	....	....
NC22707	Trachinotus ovatus	....	....	....
JQ639070	Trinectes maculatus	A....C...............	....	....
PV656769	Urophycis chuss	....	....	....
OR582684	Urophycis regia	....	....	....
MT103927	Urophycis tenuis	....	....	....
	MIFISH-U-F →	.T...................	.... ← MIFISH-U-R2	
			RIAZ-F →	.... ← RIAZ-R

S4 Fig. Primer binding sites for local marine fish species detected in this study. Consensus sequence shown at top. Only bases differing from consensus are shown. Mismatches at primer 3’ terminus predicted to significantly reduce amplification efficiency. Cunner is only species with a 3’ terminus mismatch.
