# Supplementary material for: Biomonitoring in the Anthropocene: Urban estuary environmental DNA tracks marine fish, terrestrial wildlife, and human diet
Source: PLoS One. 2026 Apr 29;21(4):e0332676. doi: 10.1371/journal.pone.0332676 (PMC13127899; doi:10.1371/journal.pone.0332676)
Supplement: S5 Fig — (PDF) [file pone.0332676.s015.pdf]

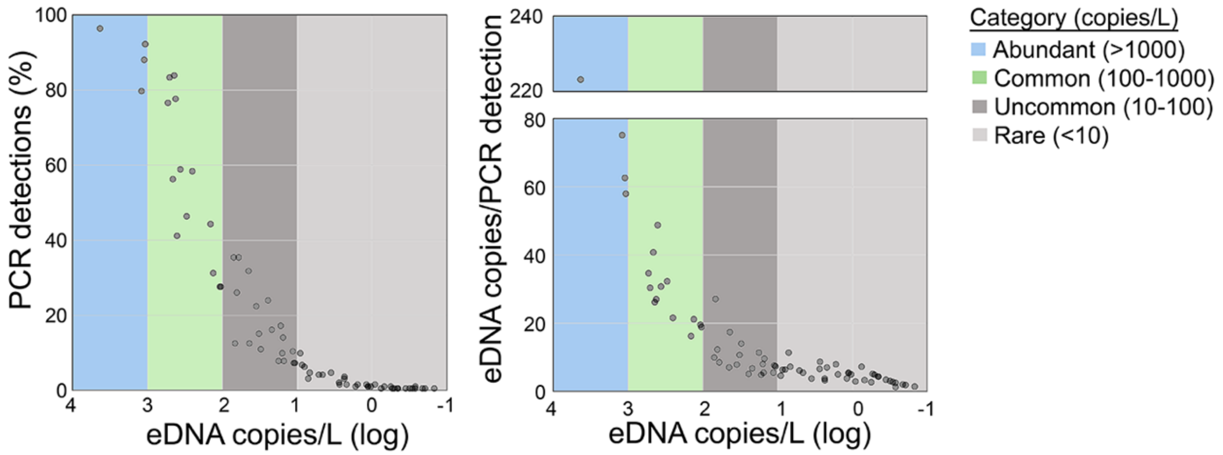

S5 Fig. Frequency of detection, copies per detection vs eDNA overall abundance. Each point represents one species. Left, frequency of PCR detection and right, eDNA copies per PCR detection, according to average eDNA abundance. Colors indicate abundance categories as in Fig 4.
