# Supplementary material for: Biomonitoring in the Anthropocene: Urban estuary environmental DNA tracks marine fish, terrestrial wildlife, and human diet
Source: PLoS One. 2026 Apr 29;21(4):e0332676. doi: 10.1371/journal.pone.0332676 (PMC13127899; doi:10.1371/journal.pone.0332676)
Supplement: S6 Fig — (PDF) [file pone.0332676.s016.pdf]

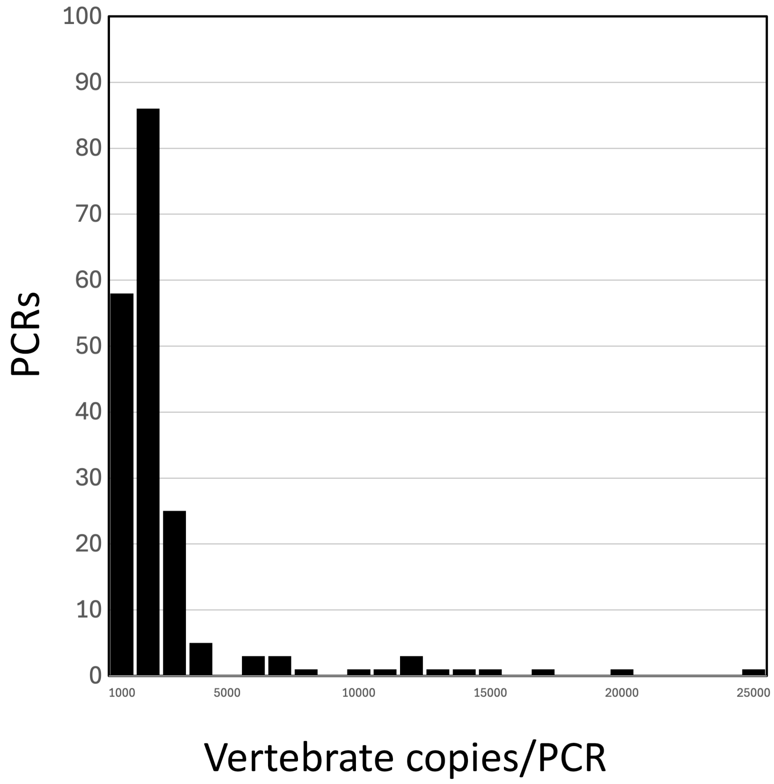

S6 Fig. Total vertebrate eDNA copies per PCR (average, 2293; range 365-24091). These appeared sufficient to detect single copy eDNA (average reads per copy per PCR, 88.1; range 3.5-325.7). Source data S3 Table.
