# Supplementary material for: Biomonitoring in the Anthropocene: Urban estuary environmental DNA tracks marine fish, terrestrial wildlife, and human diet
Source: PLoS One. 2026 Apr 29;21(4):e0332676. doi: 10.1371/journal.pone.0332676 (PMC13127899; doi:10.1371/journal.pone.0332676)
Supplement: S7 Fig — (PDF) [file pone.0332676.s017.pdf]

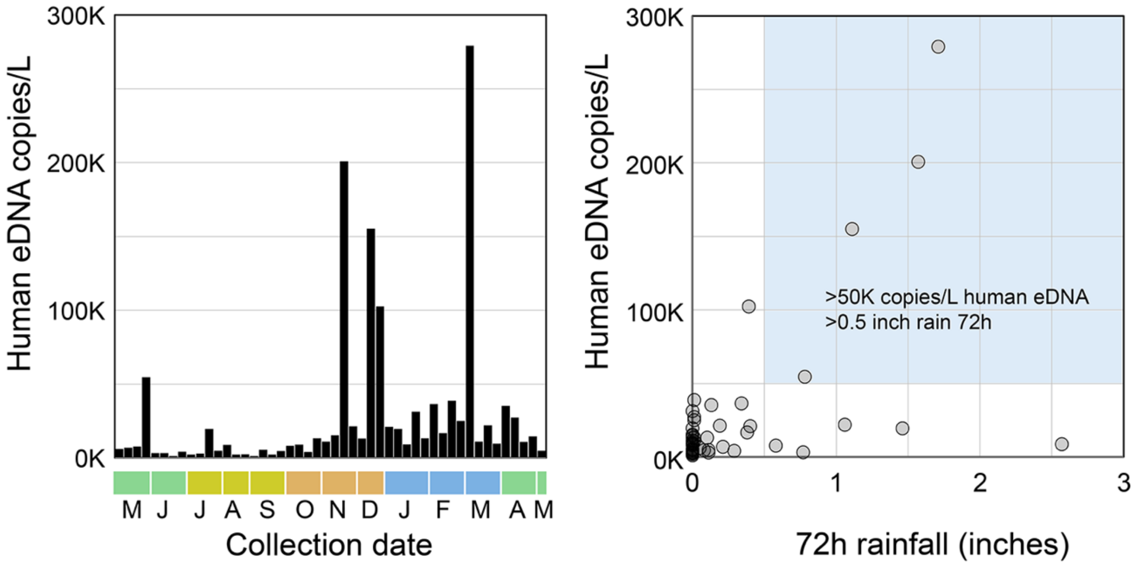

S7 Fig. Human eDNA abundance and recent rainfall. Left, each column represents one day. Right, human eDNA copies/L vs recent rainfall New York City. Recent rainfall refers to total over 72 h up to and including collecting day (collection day 0, day -1, day -2) (data from https://www.weather.gov/, New York City Central Park Station). Complete dataset in S3 Table.
