# Supplementary material for: Biomonitoring in the Anthropocene: Urban estuary environmental DNA tracks marine fish, terrestrial wildlife, and human diet
Source: PLoS One. 2026 Apr 29;21(4):e0332676. doi: 10.1371/journal.pone.0332676 (PMC13127899; doi:10.1371/journal.pone.0332676)
Supplement: S8 Fig — (PDF) [file pone.0332676.s018.pdf]

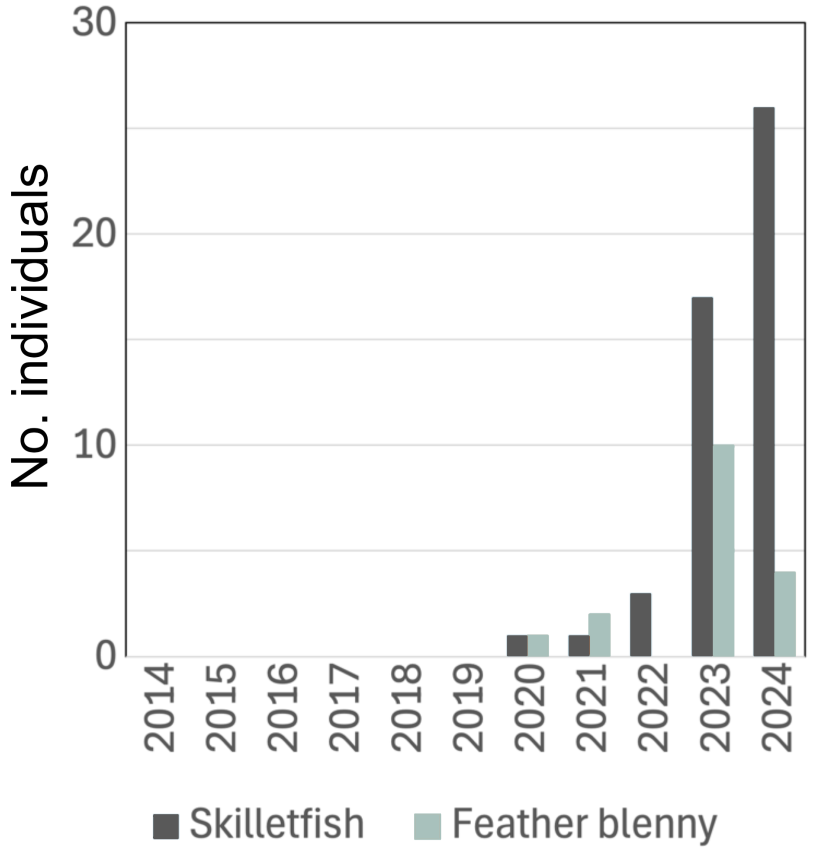

S8 Fig. Skilletfish, feather blenny captured in Hudson River Foundation Fish Survey, 2014-2024. Annual number of individuals captured in traps shown. Data from Hudson River Park Trust. Accessible at https://hudsonriverpark.org/the-park/parks-river-project/science/research/fish/
